# Development and Validation of a Highly Sensitive and Rapid LC-MS^3^ Strategy to Determine Oxcarbazepine and Its Active Metabolite in the Serum of Patients with Epilepsy and Its Application in Therapeutic Drug Monitoring

**DOI:** 10.3390/molecules27175670

**Published:** 2022-09-02

**Authors:** Zhengchao Ji, Tingting Li, Xin Zhao, Wei Ma, Yanyan Li, Jing Huang

**Affiliations:** 1 Department of Laboratory Medicine, The First Hospital of Jilin University, Jilin University, Changchun 130021, China; 2 Vascular Surgery, General Surgery Center, The First Hospital of Jilin University, Jilin University, Changchun 130021, China; 3 Department of Health Examination Center, The First Hospital of Jilin University, Jilin University, Changchun 130021, China; 4 Department of Pharmacy, The First Hospital of Jilin University, Jilin University, Changchun 130021, China

**Keywords:** oxcarbazepine, 10-hydroxycarbazepine, LC-MS^3^, validation, therapeutic drug monitoring

## Abstract

A sensitive and rapid bioanalytical method based on the LC-triple-stage fragmentation (LC-MS^3^) strategy on a hybrid triple quadrupole-linear ion trap mass spectrometer in combination with protein precipitation extraction for sample pretreatment has been developed and validated for the simultaneous determination of the antiepileptic drug oxcarbazepine (OXC) and its main active metabolite (MHD) in human serum. The separation was performed on a Waters XBridge BEH C18 column (2.5 µm, 2.1 × 50 mm) in isocratic elution with 0.1% formic acid in water and methanol (50:50, *v:v*) as the mobile phase. The run time for each sample was 2.0 min. The calibration curves ranging from 25 to 1600 ng/mL for OXC and from 0.5 to 32 μg/mL for MHD showed correlation coefficients (r) better than 0.99. All of the validation data, such as precision, accuracy and other parameters, fit the requirements of the current bioanalytical method validation guidelines. The LC-MS^3^ method for quantitation of OXC and MHD was compared with the LC-MRM based method. Passing–Bablok regression coefficients and Bland–Altman plots showed that the developed LC–MS^3^ method is a reliable method for quantitative analysis of OXC and MHD. The proposed LC-MS^3^ method was successfully applied to determine the serum concentrations of OXC and MHD to support a clinical study.

## 1. Introduction

**Figure 1:**
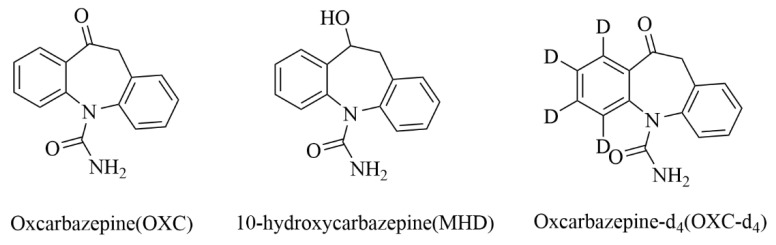
Structures of oxcarbazepine (OXC), 10-hydroxycarbazepine (MHD) and oxcarbazepine-d_4_ (OXC-d_4_, internal standard).

Oxcarbazepine (OXC; 10,11-dihydro-10-oxo-5H-dibenzo[*b,f*]azepine-5-carboxamide) (Figure 1) is a dibenzazepine carboxamide derivative with an anticonvulsant property [1]. As a second-generation antiepileptic drug, OXC is widely used for the treatment of partial-onset seizures and generalized tonic–clonic seizures in children and adults when administrated alone or in combination [2,3,4]. Although the action mechanism of OXC has not been understood completely, electrophysiological studies indicate that the agent acts primarily by promoting stabilization of hyper-excited neural membranes through the blockade of voltage-gated sodium channels in brain [5,6,7]. As a prodrug, OXC is converted into the pharmacologically active non-toxic metabolite 10-monohydroxycarbamazepine (MHD; 10-hydroxy-10,11-dihydro-5H-dibenzo[*b*,*f*]azepine-5-carboxamide) (Figure 1) [8]. Compared with OXC, MHD is present in much higher concentrations in plasma than OXC after oral administration, and then, MHD is mainly glucuronized and subsequently excreted in the urine [7,9]. The half-lives of OXC and its active metabolite (MHD) are 1.3–3.8 h and 8.8–10 h, respectively [10]. Therapeutic drug monitoring (TDM) plays an important role in the individualized treatment of epilepsy patients [11]. Serum MHD level plays a key role in the occurrence of adverse events induced by OXC. The common adverse effects of OXC include dizziness, somnolence, headache, diplopia, hyponatremia, etc. [12,13]. On the basis of these factors, overdose therapy of OXC should be avoided in its clinical application. As an important reference datum to determine the appropriate time and the dose to administer OXC, the concentration of OXC and its main active metabolite in patients’ serum could help reduce adverse effects and adjust the dose of OXC. Therefore, the TDM of OXC and MHD is critical and essential. 

To date, several bioanalytical tools have been reported for the determination of OXC and/or its active metabolite (MHD) in biological fluids, including HPLC with UV detection [14,15,16,17,18], GC/MS [19,20] and LC/MS [21,22,23,24,25]. Compared with these techniques, the LC-MS/MS method might be the best choice for the quantitation of OXC and MHD due to its higher sensitivity, specificity and stability. However, to the best of our knowledge, the LC-MS^3^ technique for the quantification of OXC and MHD in biological fluids was not discussed and studied in detail until now. The MS^3^ detection is a specific scanning mode of Q-Q-Trap tandem mass spectrometry based on a hybrid triple quadrupole-linear ion trap mass spectrometer, which could improve the excitation efficiency and scanning speed (up to 20,000 Da/s) [26,27]. In MS^3^ scanning mode, the precursor ions are first selected in Q1; then, the precursor ions are fragmented into product ions via collision-induced dissociation in a collision cell (Q2); and the product ions generated in collision cell are enriched first and then captured in Q3 (linear ion trap) [28,29]. As far as we know, we may be the first to use the LC-MS^3^ detection mode for the quantitative determination of OXC and MHD in human serum. Additionally, in this study, small volumes (15 μL) of serum samples were cleaned by a simple sample preparation of protein precipitation. The dynamic linearity ranges 25–1600 ng/mL for OXC and 0.5–32 μg/mL for MHD can cover the serum concentrations after the administration of OXC.

In the present work, we develop and validate a specific, sensitive and rapid bioanalytical method for the quantification of oxcarbazepine (OXC) and its active metabolite (MHD) in the serum of patients with epilepsy using the MS^3^ detection mode on a hybrid triple quadrupole-linear ion trap mass spectrometer. A sensitive and rapid LC-MS^3^ method for the quantification of OXC and MHD was successfully completed on the serum samples of patients with epilepsy, and the quantitative results were compared with those of the LC-MRM method for quantification of OXC and MHD. Passing–Bablok regression coefficients and Bland–Altman plots showed that there is no difference between the LC–MS^3^ and LC-MRM methods. The proposed LC-MS^3^ method was successfully applied to determine the serum concentrations of OXC and MHD to support a clinical study.

## 2. Results

### Method Validation

**Figure 2:**
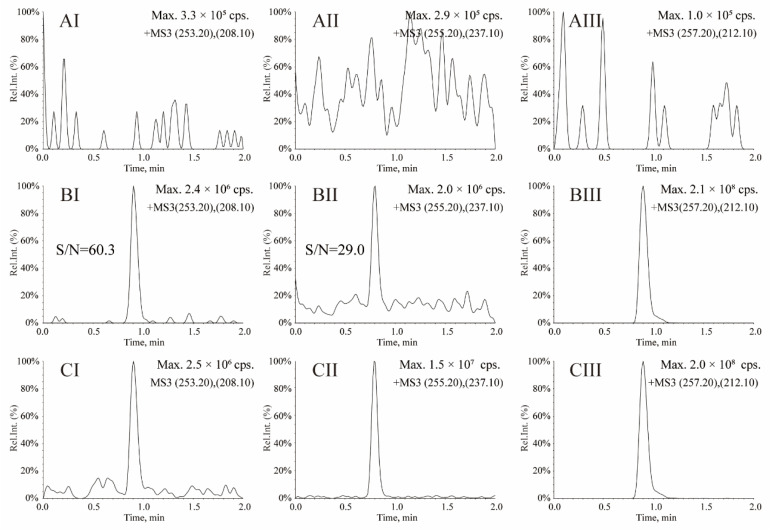
Representative LC-MS^3^ chromatograms for oxcarbazepine (**I**), 10-hydroxycarbazepine (**II**) and oxcarbazepine-d_4_ (**III**). (**A**) Blank human serum; (**B**) blank human serum spiked with oxcarbazepine at 25 ng/mL of LLOQ (**BI**), 10-hydroxycarbazepine at 0.5 µg/mL of LLOQ (**BII**) and 5.0 µg/mL of IS (**BIII**); (**C**) a serum sample from a patient with epilepsy after oral administration of oxcarbazepine.

MS detection performed in MS^3^ (linear ion trap) mode with positive polarity was used to monitor the transitions for OXC, MHD and OXC-d_4_ (IS). The MS^3^ transitions selected for the detection of OXC, MHD and OXC-d_4_ (IS) were at *m/z* 253.2→208.1→180.2, 255.2→237.1→194.1 and 257.2→212.1→184.2, respectively. For this LC-MS^3^ assay, the typical chromatograms of OXC, MHD and IS in serum are shown in Figure 2, which showed that no significant interferences in human serum were observed at the retention time. Typical retention times were 0.91, 0.76 and 0.88 min for OXC, MHD and IS, respectively. In addition, no carryover was observed among OXC, MHD and IS. For OXC, all calibration curves for LC-MS^3^ (y = 0.0397x − 0.0124, r = 0.9963) showed good linearity in the range 25–1600 ng/mL, with an LLOQ of 25 ng/mL at S/N = 60.3 (Figure 2BI). For MHD, all calibration curves for LC-MS^3^ (y = 0.231x + 0.00701, r = 0.9959) showed good linearity in the range 0.5–32 μg/mL, with an LLOQ of 0.5 μg/mL at S/N = 29.0 (Figure 2BII). For OXC and MHD, the precisions (RSD%) and accuracies (RE%) were all between −6.8% and <10.5% at all concentrations (Table 1), which indicated that the LC-MS^3^ method is reproducible and reliable. The corresponding matrix effects of OXC/MHD at three QC levels (low, medium and high) all fit the requirements of the assay, which are presented in Table 2. The recoveries for OXC/MHD at low, medium and high QC concentrations were 92.1 ± 14.9%/91.6 ± 4.8%, 112.1 ± 11.5%/93.0 ± 4.2% and 104.4 ± 3.6%/105.0 ± 8.2% (Table 2), respectively. The recoveries for OXC–d_4_ (IS) were all within 101.3 ± 6.2%. Stability data for OXC and MHD are presented in Table 3. Concentrations under the various test conditions were all within ± 15.0% of nominal concentrations, indicating that no significant degradation of OXC and MHD occurred under the storage conditions examined.

## 3. Discussion

### 3.1. Optimization of MS Conditions

**Figure 3:**
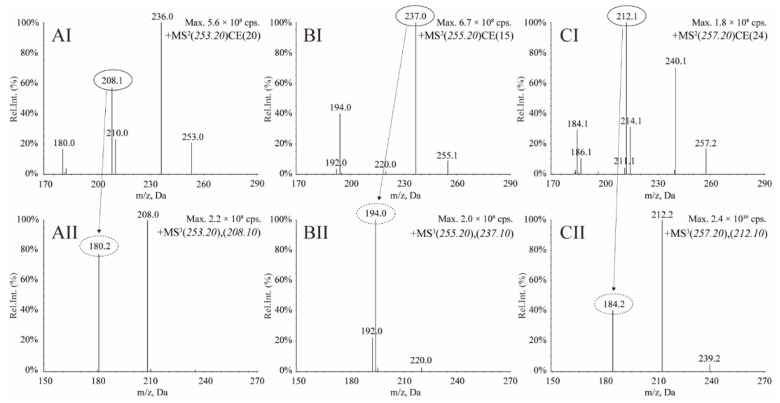
Product ion (MS^2^) and second-generation product ion (MS^3^) scans, respectively, for oxcarbazepine (**AI**,**AII**), 10-hydroxycarbazepine (**BI**,**BII**) and oxcarbazepine-d_4_ (**CI**,**CII**).

MS detection was performed in MS^3^ (linear ion trap) mode and MRM mode, and positive polarity was used to monitor the transitions for OXC, MHD and OXC-d_4_ (IS). The MRM and MS^3^ spectra for OXC, MHD and IS are shown in Figure 3. 

For OXC, the *m/z* 253.0 was adopted as a precursor ion. The MS^2^ product ions were 236.0, 210.0, 208.1 and 180.0, in which the ions at *m/z* 236.0 and 208.0 gave the intense signals. Then, the MS^2^ product ion at *m/z* 208.0 was chosen for fragmentation in the linear ion trap (MS^3^ mode), and its daughter ion (second-generation product ion) at *m/z* 180.0 gave a better response (Figure 3AI,AII). For MHD, the MS^2^ product ions for MHD at *m/z* 237.0 and 194.0 showed better signals, from which the fragment ion at *m/z* 255.1 was adopted as precursor ion and the common MS^3^ product ions at *m/z* 237.1 was selected for quantitation (Figure 3BI,BII). For OXC-d_4_, the product ions at *m/z* 212.1 was fragmented to the second-generation product ions at *m/z* 184.2, which showed a better signal (Figure 3CI,CII). Hence, in MRM mode, the MS/MS transitions selected for detection of OXC, MHD and IS were at *m/z* 253.2→208.1, 255.2→237.1 and 257.2→212.1, respectively. In MS^3^ mode, the MS^3^ transitions selected for detection of OXC, MHD and IS were at *m/z* 253.2→208.1→180.2, 255.2→237.1→194.1 and 257.2→212.1→184.2, respectively. All of the optimized MS^3^ and MRM parameters for quantitative analysis of OXC, MHD and OXC-d_4_ (IS) are shown in Table 4. The mass range scanned for second-generation product ions of OXC, MHD and IS was ± 1.0 Da. 

### 3.2. Optimization of LC Conditions

A Waters XBridge BEH C18 column (2.5 µm, 2.1 × 50 mm) and elution with 0.1% formic acid in water–methanol (50:50, *v:v*) at 0.35 mL/min was used for chromatography separation, which can provide good retention behaviors and peak shapes of OXC, MHD and IS. An isocratic elution with the ratio of organic phase-aqueous phase (50:50, *v:v*) could eliminate the matrix effects and reduce the carryover. Under the optimized conditions, the retention times of OXC, MHD and IS were 0.91, 0.76 and 0.88 min, respectively. Finally, isocratic elution with 0.1% formic acid in water and methanol (50:50, *v:v*) at 40 °C gave the best results (Figure 2).

### 3.3. Optimization of Sample Preparation

Based on advantages such as convenience, simplicity and rapidity, protein precipitation with methanol was employed for sample processing, which can reduce the negative effects of the interfering substances present in a serum sample. A total of 15 μL of human serum sample was mixed with 20 μL IS solution (5 μg/mL) and 250 μL methanol to precipitate proteins. Then, the test tube were vortex-mixed for 60 s on a shaker, followed by centrifugation at 15,000 rpm and 4 °C for 5 min, and the upper clear solution layer (solution A) was used for quantitative analysis of OXC. To 25 µL of the upper clear solution layer, 600 µL of water was vortex-mixed for 1 min, and the diluted supernatant (solution B) was used for the determination of MHD. Finally, a total of 2 μL of solution A or solution B was injected into the LC-MS system before analysis. LLOQs of 25 ng/mL for OXC and 0.5 µg/mL for MHD were sufficient in this study. Additionally, the LLOQs of OXC and MHD in this assay could be easily reduced by using more serum or less dilution or more injection volume in LC eluent. 

### 3.4. Method Comparison and Clinical Application

Compared with the LC-MS^3^ assay, A LC-MS method using MRM transitions at *m/z* 253.2→208.1 for OXC, *m/z* 255.2→237.1 for MHD and *m/z* 257.2→212.1 for IS was optimized. For the LC-MRM method, the validation data of OXC and MHD including precision, accuracy, matrix effect, recovery and other parameters all fit the requirements of current bioanalytical method validation guidelines, which were shown in Tables S1–S3 (Supplementary Materials). 

**Figure 4:**
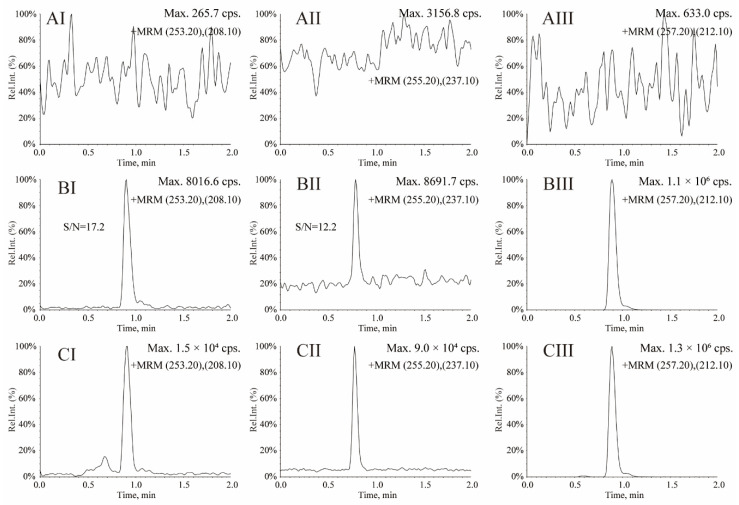
Representative LC-MRM chromatograms for oxcarbazepine (**I**), 10-hydroxycarbazepine (**II**) and oxcarbazepine-d_4_ (**III**). (**A**) Blank human serum; (**B**) blank human serum spiked with oxcarbazepine at 25 ng/mL of LLOQ (**BI**), 10-hydroxycarbazepine at 0.5 µg/mL of LLOQ (**BII**) and 5.0 µg/mL of IS (**BIII**); (**C**) a serum sample from a patient with epilepsy after oral administration of oxcarbazepine.

The typical chromatograms of LC-MRM assay for OXC, MHD and IS in serum are shown in Figure 4, which showed that no significant interferences in human serum were observed at the retention time. The chromatograms of OXC at 25 ng/mL (LLOQ) obtained by the LC-MS^3^ and LC-MRM methods are shown in Figure 2BI and Figure 4BI, respectively. The chromatograms of MHD at 0.5 µg/mL (LLOQ) obtained by the LC-MS^3^ and LC-MRM methods are shown in Figure 2BII and Figure 4BII, respectively. For MS^3^ acquisition, the response signals for OXC and MHD are 2.4 × 10^6^ cps (counts per second) with S/N = 60.3 at 25 ng/mL and 2.0 × 10^6^ cps with S/N = 29.0 at 0.5 µg/mL (Figure 2BI,BII), respectively. For LC-MRM acquisition, the response signals for OXC and MHD are 8016.6 cps with S/N = 17.2 at 25 ng/mL and 8691.7 cps with S/N = 12.2 at 0.5 µg/mL (Figure 4BI,BII), respectively. Obviously, compared with MRM transition, the sensitivities and S/Ns of OXC and MHD in MS^3^ scan are significantly improved due to the additional fragmentation step.

**Figure 5:**
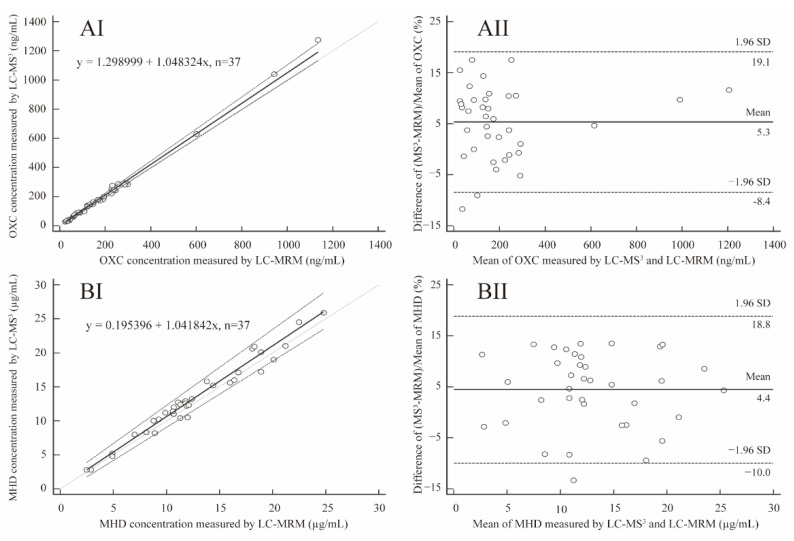
Comparison of the LC-MS^3^ assay and LC-MRM method. (**I**) Passing–Bablok plots for the correlation analysis between LC-MS^3^ and LC-MRM concentrations for oxcarbazepine (**AI**) and 10-hydroxycarbazepine (**BI**). Solid black lines represent Passing–Bablok regression. (**II**) Bland–Altman plot for the comparison between LC-MS^3^ and LC-MRM concentrations for oxcarbazepine (**AII**) and 10-hydroxycarbazepine (**BII**). Points between the dashed lines indicate sample pairs that are within the ±1.96 SD acceptance range.

The validated LC-MS^3^ method was applied to monitor the OXC/MHD concentrations of serum samples obtained from patients with epilepsy, following OXC treatment with a daily oral dose. The concentrations of OXC/MHD in 37 human serum samples were obtained by LC-MS^3^ and LC-MRM methods, which were shown in Table S4 (Supplementary Materials). A comparison of the serum concentrations of OXC/MHD measured by LC-MS^3^ and LC-MRM methods is shown in Figure 5. The results from these two methods of OXC/MHD measurements were acceptably close; had no constant bias; and had no proportional bias, as expressed in the Passing and Bablok regression equations of y= 1.298999 (95% CI, −3.5937 to 4.8000) + 1.048324 (95% CI, 1.0000 to 1.0999)x for OXC and y = 0.195396 (95% CI, −0.6733 to 1.0834) + 1.041842 (95% CI, 0.9733 to 1.1188)x for MHD (Figure 5AI,BI). Bland–Altman plots showed that the differences in OXC and MHD concentration measured by LC-MS^3^ and LC-MRM was 5.3% (95% LoA, −8.4% to 19.1%) and 4.4% (95% LoA, −10.0% to 18.8%) (Figure 5AII,BII), respectively. The differences in OXC/MHD were evenly distributed on both sides of the mean; 94.6% (35/37) of the OXC sample pairs and 97.3% (36/37) of the MHD sample pairs had a maximal concentration deviation of ±1.96 SD (Figure 5AII,BII), indicating that the LC-MS^3^ strategy is a valuable and reliable method for drug monitoring of OXC/MHD.

## 4. Materials and Methods

### 4.1. Reagents and Chemicals

Oxcarbazepine (OXC), 10-hydroxycarbazepine (MHD) and oxcarbazepine-d_4_ (OXC-d_4_, IS) were purchased from National Institutes for Food and Drug Control (Beijing, China), Shanghai Pufen Biotechnology Co. Ltd. (Tianjin, China) and Toronto Research Chemicals (Toronto, Canada) and were presented in Figure 1. Methanol and formic acid were purchased from Fisher Scientific (Fair Lawn, NJ, USA). Deionized water (18.2 MΩ·cm) was purchased from a Millipore system (Millipore, Bedford, MA, USA). All chemicals and reagents were of HPLC grade.

### 4.2. Chromatographic and Mass Spectrometric Conditions

Liquid chromatography was performed on a LC-20 AD series HPLC system equipped with two binary pumps, a thermostatically controlled column compartment set at 40 °C and an auto sample manager maintained at 4 °C (Shimadzu Corporation, Kyoto, Japan). The separation was performed on a Waters XBridge BEH C18 column (2.5 µm, 2.1 × 50 mm) in isocratic elution with 0.1% formic acid in water and methanol (50:50, *v:v*). The flow rate and the injection volume were set at 0.35 mL/min and 2 µL, respectively. 

Tandem mass spectrometry was carried out on a Qtrap 5500 mass spectrometer (AB SCIEX, Ontrario, Canada) equipped with electrospray ionization in the positive ion mode. Linear ion trap (MS^3^) mode and Multiple Reaction Monitoring (MRM) mode were employed for the data acquisition of OXC, MHD and OXC-d_4_ (internal standard, IS). The optimized MS parameters were as follows: ionspray needle voltage 5500 V; heater gas temperature 450 °C; curtain gas 30 psi; nebulizer gas 50 psi; and heater gas 50 psi. Other operation conditions of MS^3^ transitions and MRM transitions optimized parameters for quantitative analysis of OXC, MHD and OXC-d_4_ (IS) are presented in Table 4.

### 4.3. Preparation of Stock Solutions, Calibration Standards and Quality Control (QC) Samples

The primary standard stock solutions of OXC (150 μg/mL) and MHD (1 mg/mL) were prepared separately by dissolving each standard in methanol and then mixed with a volume ratio of 1:1 to make a mixed stock solution. The calibration standards were obtained by diluting the mixed stock solutions with blank human serum to obtain concentrations of 25, 50, 100, 200, 400, 800 and 1600 ng/mL for OXC and 0.5, 1, 2, 4, 8, 16 and 32 μg/mL for MHD. Quality control (QC) samples were prepared independently in the same way. Quality control (QC) samples were fixed at the different concentrations of 50 ng/mL (low, LQC), 200 ng/mL (medium, MQC) and 800 ng/mL (high, HQC) for OXC and at the different concentrations of 1 μg/mL (low, LQC), 4 μg/mL (medium, MQC) and 16 μg/mL (high, HQC) for MHD, respectively. An IS stock solution of OXC-d_4_ (1 mg/mL) was prepared in methanol and diluted to 5 μg/mL with methanol:water (50:50, *v:v*). All solutions were stored at −20 °C until use.

### 4.4. Serum Sample Pretreatment

Frozen human serum samples were thawed at room temperature. A total of 15 μL of human serum sample was mixed with 20 μL of IS solution (5 μg/mL) and 250 μL of methanol to precipitate proteins. Then, the test tubes were vortex-mixed for 60 s on a shaker, followed by centrifugation at 15,000 rpm and 4 °C for 5 min, and the upper clear solution layer (solution A) was used for quantitative analysis of OXC. To 25 µL of the upper clear solution layer, 600 µL of water was vortex-mixed for 1 min, and the diluted supernatant (solution B) was used for the determination of MHD. Finally, a total of 2 μL of solution A or solution B was injected into the LC-MS system before analysis. The calibration standard samples and QC samples were prepared in the same way as the serum samples.

### 4.5. Method Validation for the Quantitation of OXC and MHD in Human Serum

The method validation, including specificity, linearity, accuracy, precision, recovery and stability, was conducted according to the bioanalytical method validation guidelines of the US Food and Drug Administration (FDA) and the Chinese Pharmacopeia (215 edition) [30,31]. The details for assay validation are described in the Supplementary Materials.

### 4.6. Clinical Application and Ethics

In order to verify the applicability of the developed LC-MS^3^ assay, 37 real human serum samples obtained from patients with epilepsy (weight: 45–70 kg, age: 14–72 years old, sex: 24 male, 13 female) under treatment at the First Hospital of Jilin University were analyzed. All epileptic patients were treated with oxcarbazepine only. The therapeutic regimen is 600 mg/day for each person. OXC/MHD stead-state trough concentrations were collected from 37 patients with epilepsy who received OXC for at least 1 week.

The study was approved by the Ethics Committee of the First Hospital of Jilin University (Ethical Approval Number: 2021-522; date: 8 January 2021). The clinical applicability of the LC-MS^3^ method was assessed by an analysis of OXC and MHD in human serum samples from patients referred to our therapeutic drug monitoring laboratory to monitor OXC and MHD routinely.

### 4.7. Data Acquisition and Processing

Data acquisition, data processing and graphic presentation were carried out using Analyst 1.6.3 software (AB SCIEX, Foster City, CA, USA), Microsoft 2007 (Microsoft, Bellevue, WA, USA) and MedCalc Version 15.2.2 (MedCalc Software, Mariakerke, Belgium), respectively. Passing–Bablok regression and Bland–Altman analysis were used to assess the agreement between the serum concentrations of OXC and MHD calculated from the LC-MS^3^ data and LC-MRM data. The assay agreement was considered sufficient if difference were within ±1.96 SD of the mean difference for ≥67% of the sample pairs. The serum concentrations of OXC and MHD were calculated based on LC-MS^3^ and LC-MRM data, respectively.

## 5. Conclusions

A highly reliable and selective LC-MS^3^ bioassay for the quantitation of OXC and its main active metabolite (MHD) in human serum has been developed and validated. The volume of serum for sample preparation is only 15 µL, and the LC-MS^3^ assay is free of matrix interference and improves the sensitivity and S/N ratio compared with quantitation by conventional LC-MS/MS with MRM. To the best of our knowledge, this is the first study using a LC-MS^3^ technique for the quantification of OXC and MHD in human serum and its application to a therapeutic drug monitoring. This work is also a proof-of-concept study of LC-MS^3^ for the quantitative analysis of compounds in biological samples.

## Figures and Tables

**Table 1:** Precision and accuracy of oxcarbazepine (OXC) and 10-hydroxycarbazepine (MHD) from LC-triple-stage fragmentation (MS^3^) method.

Compound	Spiked Concentration (µg/mL)	Precision (RSD, %)		Accuracy (RE, %)	
		Intra-day (n = 6)	Inter-day (3 days, n = 18)	Intra-day (n = 6)	Inter-day (3 days, n = 18)
OXC	0.05	8.3	7.0	8.6	4.9
	0.2	7.0	6.1	−6.8	−2.8
	0.8	1.4	0.8	1.2	0.8
MHD	1	10.5	10.2	8.5	0.5
	4	8.5	9.2	7.9	−0.5
	16	9.5	9.3	7.3	0.7

Abbreviations: OXC, oxcarbazepine; MHD, 10-hydroxycarbazepine.

**Table 2:** Matrix effect and recovery of oxcarbazepine (OXC) and 10-hydroxycarbazepine (MHD) from LC-triple-stage fragmentation (MS^3^) method.

Compound	Spiked Concentration (µg/mL)	Matrix Effect (%) Mean ± SD (*n* = 6)	Recovery (%) Mean ± SD (*n* = 6)
OXC	0.05	92.7 ± 6.4	92.1 ± 14.9
	0.2	99.8 ± 10.4	112.1 ± 11.5
	0.8	101.8 ± 6.8	104.4 ± 3.6
MHD	1	99.1 ± 10.0	91.6 ± 4.8
	4	96.1 ± 1.0	93.0 ± 4.2
	16	103.1 ± 3.9	105.0 ± 8.2

Abbreviations: OXC, oxcarbazepine; MHD, 10-hydroxycarbazepine.

**Table 3:** Stability of oxcarbazepine (OXC) and 10-hydroxycarbazepine (MHD) under various storage conditions (data are mean ± SD, %, n = 3).

Compound	Nominal Concentration (µg/mL)	Long Term −80 °C	Short Term	Freeze–Thaw	Post-Preparative
OXC	0.05	90.5 ± 6.9	97.5 ± 7.4	92.7 ± 8.2	95.0 ± 5.7
	0.2	93.8 ± 5.3	91.3 ± 5.1	89.3 ± 1.0	90.5 ± 2.7
	0.8	99.6 ± 1.8	92.4 ± 2.0	98.9 ± 1.7	97.5 ± 0.9
MHD	1	95.6 ± 9.3	98.6 ± 12.1	96.9 ± 4.7	97.4 ± 7.1
	4	92.5 ± 4.8	98.9 ± 0.95	100.0 ± 7.5	10.9 ± 2.4
	16	104.4 ± 2.5	97.7 ± 6.6	96.0 ± 9.9	96.0 ± 9.9

Abbreviations: OXC, oxcarbazepine; MHD, 10-hydroxycarbazepine.

**Table 4:** Optimized parameters for the quantitation of oxcarbazepine (OXC) and 10-hydroxycarbazepine (MHD) using triple-stage fragmentation (MS^3^) and multiple reaction monitoring (MRM) modes.

Parameters	MS^3^			MRM		
	OXC	MHD	OXC-d_4_ (IS)	OXC	MHD	OXC-d_4_ (IS)
MS^3^ transitions/MRM transitions (*m/z*)	253.2→208.1→180.2	255.2→237.1→194.1	257.2→212.1→184.2	253.2→236.1	255.2→237.1	257.2→240.1
Declustering potential (V)	100	60	100	100	60	100
Collision energy (eV)	23.5	16.1	26.3	23.5	16.1	26.3
AF2 (V)	0.10	0.10	0.10	/	/	/
Scan rate (Da/s)	20000	20000	20000	/	/	/
LIT fill time (ms)	20	20	20	/	/	/
Excitation time (ms)	25	25	25	/	/	/

Abbreviations: OXC, oxcarbazepine; MHD, 10-hydroxycarbazepine; OXC-d_4_, oxcarbazepine-d_4_; IS, internal standard. AF2: Excitation Energy.

## Data Availability

The data presented in this study are available from the corresponding author upon request.

## Supplementary Materials

The following supporting information can be downloaded at https://www.mdpi.com/article/10.3390/molecules27175670/s1, Table S1: Precision and accuracy of oxcarbazepine (OXC) and 10-hydroxycarbazepine (MHD) from LC-multiple reaction monitoring (MRM) method. Table S2: Matrix effect and recovery of oxcarbazepine (OXC) and 10-hydroxycarbazepine (MHD) from LC-multiple reaction monitoring (MRM) method. Table S3: Stability of oxcarbazepine (OXC) and 10-hydroxycarbazepine (MHD) under various storage conditions from LC-multiple reaction monitoring (MRM) method (data are mean ± SD, %, *n* = 3); Table S4: Concentrations of Oxcarbazepine (OXC) and 10-hydroxycarbazepine (MHD) in 37 human serum samples analyzed by LC-triple-stage fragmentation (MS^3^) and LC-multiple reaction (MRM) methods.

## Sample Availability

Samples of the compounds are available from the authors.
