# Supplementary material for: Development and Validation of a Highly Sensitive and Rapid LC-MS3 Strategy to Determine Oxcarbazepine and Its Active Metabolite in the Serum of Patients with Epilepsy and Its Application in Therapeutic Drug Monitoring
Source: Molecules. 2022 Sep 2;27(17):5670. doi: 10.3390/molecules27175670 (PMC9457704; doi:10.3390/molecules27175670)
Supplement: Supplementary file 1 [file molecules-27-05670-s001.zip › molecules-1844703-Supplementary.pdf]

Development and Validation of a Highly Sensitive and Rapid LC-MS^3 Strategy to Determine Oxcarbazepine and its Active Metabolite in the Serum of Patients with Epilepsy and its Application in Therapeutic Drug Monitoring

Supplementary Materials

Zhengchao Ji [1†], Tingting Li [2†], Xin Zhao [3], Wei Ma [4], Yanyan Li [1*], Jing Huang [1*]

1 Department of Laboratory Medicine, The First Hospital of Jilin University, Jilin University, Changchun, 130021, China

2 Vascular surgery, General Surgery Center, The First Hospital of Jilin University, Jilin University, Changchun, 130021, China

3 Department of Health Examination Center, The First Hospital of Jilin University, Jilin University, Changchun, 130021, China

4 Department of Pharmacy, The First Hospital of Jilin University, Jilin University, Changchun, 130021, China

* Correspondence: yanyanli@jlu.edu.cn (Y.L.); huangj@jlu.edu.cn (J. H.).

† These authors contributed equally to this work.

1. Method validation for the quantitation of clonazepam in human serum

The assay validation was performed according to the bioanalytical method validation guideline of the U.S. Food and Drug Administration (FDA). The procedure for assay validation is detailed as follows:

a) Selectivity

The selectivity of the method was tested by comparing chromatograms of six individual drug-free human serum samples to test the interference at retention time of the peaks at LLOQ of analytes and IS using the sample preparation procedure and chromatographic-MS method. Usually, it is generally acceptable that the responses of interfering components in the blank are within 20% of LLOQ and 5% for the responses of internal standards.

b) Carry-over effect

Carry-over effect was investigated by comparing chromatograms of six individual drug-free human serum samples to examine the interference at retention time of the peaks at LLOQ of analyte and IS using the sample preparation procedure and chromatographic-MS method. Carry over interference was evaluated by analyzing three blank samples after the calibration standard with the highest concentration (ULOQ). The carry-over effect of these blank samples should be within 20% of the lowest calibrator.

c) Linearity and lower limit of quantification (LLOQ)

The quantitation range of clonazepam was chosen in terms of the therapeutic window. The calibration curve was constructed by plotting the relative peak area ratio of the chemical analyte to IS (y) versus the actual concentration ratio of analyte to IS (x) using a linear weighted least squares model ($1/x^2$) at seven different concentrations. The acceptance criteria should be within ± 15% of theoretical concentrations (± 20% at LLOQ). The lower limit of quantification (LLOQ) is defined as the lowest concentration of the calibration curve which could be validated with an acceptable precision and accuracy.

d) Precision and accuracy

The intra- and inter-day precision and accuracy were investigated by analyzing six replicates at three different QC levels (low, medium and high). The precision and accuracy were presented as RSD and RE, respectively. The RSD of precision should not exceed ± 15% of nominal concentrations (± 20% at LLOQ). The RE of accuracy should not exceed ± 15% of theoretical concentrations (± 20% at LLOQ).

e) Matrix effect and extraction recovery

The matrix effect (ME) for analyte was evaluated by comparing the peak area ratio of the analyte to IS in the presence of matrix (n = 6) with the peak area ratio of the analyte to IS in absence of matrix (pure solution of the analyte and IS). Three concentration levels (QC low, QC medium and QC high) were examined. The overall CV of the IS-normalized ME between the six batches of the same matrix should be less than 15%. Extraction recovery was tested for analyte at QC low, QC medium and QC high concentrations in six lots of human serum. The recovery of analyte was calculated by comparing the peak area of blank matrix samples spiked before and after extraction.

f) Stability

The stability of spiked human serum was assessed using six replicates at three QC levels (QC low, QC medium and QC high). The following stability studies should be conducted: a) Long-term stability (storage of QC sample at −80°C for 30 days), b) Short-term stability (storage of QC sample at room temperature for 8 h), c) Freeze/thaw stability (three freeze/thaw cycles −20 °C to room temperature), d) post-preparative storage stability (autosampler temperature at 4 °C for 24 h). The accuracy (% nominal) at each level should be within ± 15 %.

2. Table lists

Table S1. Precision and accuracy of oxcarbazepine (OXC) and 10-hydroxycarbazepine (MHD) from LC-multiple reaction monitoring (MRM) method.

Compound	Spiked concentration (μg/mL)	Precision (RSD, %)		Accuracy (RE, %)	
		Intra-days (n = 6)	Inter-days (3 days, n = 18)	Intra-days (n = 6)	Inter-days (3 days, n = 18)
OXC	0.05	7.4	6.1	9.5	5.6
	0.2	7.0	6.1	-5.8	11.9
	0.8	1.4	1.7	3.3	1.6
MHD	1	6.9	8.1	8.9	1.8
	4	4.5	3.6	-5.8	-4.5
	16	5.1	6.4	6.9	2.2

Abbreviations: OXC, oxcarbazepine; MHD, 10-hydroxycarbazepine.

Table S2. Matrix effect and recovery of oxcarbazepine (OXC) and 10-hydroxycarbazepine (MHD) from LC-multiple reaction monitoring (MRM) method.

Compound	Spiked concentration (μg/mL)	Matrix effect (%) Mean ± SD (n = 6)	Recovery (%) Mean ± SD (n = 6)
OXC	0.05	101.6 ± 5.7	103.2 ± 1.9
	0.2	102.5 ± 4.3	99.0 ± 3.1
	0.8	98.1 ± 1.3	96.4 ± 6.5
MHD	1	99.2 ± 2.0	95.6 ± 4.8
	4	99.8 ± 10.1	96.6 ± 3.8
	16	103.3 ± 5.7	100.7 ± 8.4

Abbreviations: OXC, oxcarbazepine; MHD, 10-hydroxycarbazepine.

Table S3. Stability of oxcarbazepine (OXC) and 10-hydroxycarbazepine (MHD) under various storage conditions from LC-multiple reaction monitoring (MRM) method (data are mean ± SD, %, n = 3).

Compound	Nominal Con. (μg/mL)	Long term -80 °C	Short term	Freeze-thaw	Post-preparative
OXC	0.05	98.0 ± 3.9	94.2 ± 8.3	92.7 ± 2.8	95.5 ± 8.5
	0.2	92.5 ± 3.5	89.3 ± 2.5	92.2 ± 2.8	91.7 ± 2.5
	0.8	95.2 ± 1.6	98.9 ± 4.1	96.2 ± 1.2	92.1 ± 1.8
MHD	1	99.1 ± 9.2	89.8 ± 4.2	96.5 ± 8.8	93.3 ± 3.1
	4	98.2 ± 7.3	95.8 ± 4.8	93.2 ± 0.5	95.1 ± 1.2
	16	104.6 ± 1.6	103.3 ± 6.0	97.7 ± 2.5	95.8 ± 6.1

Abbreviations: OXC, oxcarbazepine; MHD, 10-hydroxycarbazepine.

Table S4. Concentrations of oxcarbazepine (OXC) and 10-hydroxycarbazepine (MHD) in 37 human serum samples analyzed by LC-triple-stage fragmentation (MS^3) and LC-multiple reaction (MRM) methods.

Sample ID	OXC (ng/mL)			MHD (μg/mL)		
	MS^3	MRM	MS^3/MRM%	MS^3	MRM	MS^3/MRM%
S1	283.7	298.8	94.9	20.6	18.1	113.8
S2	293.5	290.5	101.0	12.9	11.8	109.3
S3	147.5	141.1	104.5	21	21.2	99.1
S4	252.6	227.6	111.0	17.1	16.8	101.8
S5	145.1	136.1	106.6	10.2	9.26	110.2
S6	60.1	57.91	103.8	2.8	2.88	97.2
S7	98.0	107.3	91.3	8.3	8.1	102.5
S8	35.7	40.15	88.9	5.2	4.9	106.1
S9	181.9	189.3	96.1	11.2	9.9	113.1
S10	138.8	120.2	115.5	20.1	18.9	106.3
S11	43.6	44.2	98.6	4.8	4.9	99.0
S12	88.6	88.6	100.0	11.1	10.6	104.7
S13	221.3	226.1	97.9	20.9	18.3	114.2
S14	157.3	145.3	108.3	12.6	11.3	111.5
S15	38.2	35.2	108.5	17.2	18.9	91.0
S16	171.6	176	97.5	2.8	2.5	112.0
S17	283.3	285.3	99.3	19	20.1	94.5
S18	286.6	258	111.1	12.6	11.8	106.8
S19	150.2	146.4	102.6	13.2	12.4	106.5
S20	87.5	73.42	119.2	8.2	8.9	92.2
S21	92.0	83.53	110.1	12.7	11.1	114.4
S22	241.2	243.9	98.9	16	16.4	97.6
S23	274.7	230.5	119.2	15.8	13.8	114.5
S24	145.2	131.7	110.3	15.6	16.0	97.5
S25	246.6	237.5	103.8	15.2	14.4	105.6
S26	163.1	146.2	111.6	12.4	11.3	109.7
S27	629.1	600.5	104.8	10.0	8.8	113.6
S28	1039.0	942.8	110.2	25.9	24.8	104.4
S29	199.2	194.6	102.4	24.5	22.5	108.9
S30	28.5	24.4	116.8	11.0	10.7	102.8
S31	66.5	61.7	107.8	10.4	11.3	92.0
S32	179	168.7	106.1	12	10.7	112.1
S33	27.7	25.2	109.9	11.4	10.6	107.5
S34	73.2	64.7	113.1	8.0	7.0	114.3
S35	131.7	121.3	108.6	10.5	12	87.5
S36	33.1	30.3	109.2	12.2	11.9	102.5
S37	1275.0	1135.0	112.3	12.3	12.1	101.7
